# Assessing the Impact of an Original Soft Communicative Robot in a Nursing Home in Japan: Will Softness or Conversations Bring more Smiles to Older People?

**DOI:** 10.1007/s12369-021-00815-4

**Published:** 2021-08-07

**Authors:** Kazuko Obayashi, Naonori Kodate, Shigeru Masuyama

**Affiliations:** 1 Faculty of Healthcare Management, Nihon Fukushi University, Mihama, Japan; 2 Social Welfare Corporation Tokyo Seishin-kai, Nishitokyo, Japan; 3 Universal Accessibility and Ageing Research Centre, Nishitokyo, Japan; 4 School of Social Policy, Social Work and Social Justice, University College Dublin, Hanna Sheehy Skeffington Building, Belfield, Dublin 4, D04 N9Y1 Ireland; 5 Institute for Future Initiatives, University of Tokyo, Tokyo, Japan; 6 Public Policy Research Center, Hokkaido University, Sapporo, Japan; 7 La Fondation France-Japon, L’ École des hautes études en sciences sociales, Paris, France; 8 Traveler’s Medical Center, Tokyo Medical University, Tokyo, Japan

**Keywords:** Socially assistive robot, Eldercare, Communication, User-centered design, Human–robot interaction, Technology assessment

## Abstract

It has been reported that robotics-aided care can contribute to enhancing older people’s social participation and quality of life in nursing homes, while simultaneously reducing the burden on care professionals at nighttime. Due to increasing demand for social care and the relative workforce shortage, it is likely that a greater number and variety of robots will be introduced and implemented in the future. While the benefits of applying robots and assistive technologies are recognized, the current limitations and weaknesses have also been identified. One of these is the difficulty associated with a user-centered design, involving older adults with impaired cognitive and sensory abilities in nursing homes. In order to overcome this challenge, a project was carried out to develop a soft and compact bedside communication robot with an input/output device, connected to existing technologies (e.g. monitoring camera, biological sensor). Drawing on the principle of *gemba* (deference to frontline professionals’ experience, expertise and skills), users’ feedback was reflected in the iterative steps of robot development. The original soft and communicative robot was introduced and its effectiveness was tested by measuring older people’s reactions and changes in their behaviors and engagement levels. The article reports the development process and results of a small-scale evaluation study, comparing the impact of this original soft-type robot with and without its communicative functions. The human–robot interactions were captured on video, and the analysis revealed that while communicative robots reduced the psychosocial burden on older adults, positive emotional, verbal, visual and behavioral engagement was generated with the help of the non-verbal plush toy.

## Introduction

The global population aged 60 years or over reached 962 million in 2017, more than twice the population reported in 1980. The pace of aging is accelerating across the world and the number of older people is projected to reach more than 2 billion by 2050. Accordingly, the number of people affected by dementia has been on the increase, and the current number (approximately 47 million, as of 2018) is estimated to reach 75 million by 2030 [1]. The World Health Organization (WHO) published a report in 2015 [2], which outlined a framework for action to foster healthy aging based on the new concept of functional ability. In order to support healthy aging and meet the increasing demand (e.g. shortage of workforce), manufacturers and policymakers across the globe are emphasizing development and production of more efficient and effective assistive technologies (ATs) including care robots. Furthermore, the recent global pandemic has affected the lives of older people particularly harshly, and reinforced the view that building capacity in health systems by utilizing technologies should be one of the public policy priorities.

Research shows that older people’s acceptance of care robots can be affected by the robot’s appearance, materials and functionalities [3, 4]. It is suggested that even younger users prefer soft and cuddly robots over those with a cold, hard shell [5]. However, thus far usability, user-friendliness and outcome evaluation of these care robots have rarely been tested from the end-users’ point of view [6]. Moreover, in long-term care settings, it is not uncommon that some older users cannot verbally express their needs. To address these weaknesses, a user-centered design has been introduced for socially assistive robots (SARs) and promoted in some countries [7–9]. Globally, the Responsible Research and Innovation (RRI) framework has been proposed and used [9]. In Japan in recent years, users’ needs have been explored and identified by several key players such as the Japanese Association of Occupational Therapists and the Association of Technical Aids (established as a public interest incorporated foundation in 1987) [10]. These efforts are primarily targeted at understanding the ’needs’ of users (care recipients and caregivers) so that manufacturers, funding bodies and care providers can develop their ’seeds’ and deliver technological aids. Despite these efforts, user involvement is still a rare occurrence. This is partly because hurdles to a participatory approach remain high when the users include older adults with impaired ability. Under such circumstances, caregivers’ inputs as proxy for older people’s voice become highly important and valuable, although it should be acknowledged that this is not the same as public and patient involvement.

In the Japanese context, with the concept of *gemba* (‘actual site/field’ in Japanese), professional expertise, experience and skills have been highly valued. Originating in the manufacturing industry, gemba has been adopted in healthcare, alongside the more widely-known concept of *kaizen* (‘improvement’ in Japanese), even outside Japan [10, 11]. Guided by this bottom-up process and principle, frontline care professionals proactively identified the needs of care recipients, incorporated changes into the development of SARs, and received feedback from the care recipients. We hypothesized that by making SARs more user-friendly and accessible, better outcomes and a higher quality of life would result. In order to examine this hypothesis, research was conducted to assess the impact of using such SARs on older people.

Concerning the outcome evaluation of using existing SARs, previous studies highlighted positive impacts on older people [12–20]. Examples from the previous studies include the positive impact of SARs on older people’s activity and social participation [4, 21] and on older adults with moderate dementia [22, 23]. When using a soft tactile robot, the non-verbal type can elicit reactions in older people with advanced dementia [22]. On the other hand, when using verbal type SARs, research indicated that other features such as input/output devices and sound volume need adjustments and improvements in order for the care system to utilize the equipment effectively [22]. Further development of soft and cuddly SARs and the evaluation of their impact on older people in residential care settings was deemed necessary to advance the science behind the effectiveness of these care robots.

Against this background, this project was launched to develop and assess a bedside communication soft robot with an input/output device, connected to a monitoring camera and biological sensor, while reflecting the needs of direct users (care professionals and care recipients). Given our previous research, the purpose was to design and develop a soft robot [23] that can be used both as a non-verbal and verbal companion plush toy, as well as a socially interactive communication tool. The effectiveness of the SAR produced was also tested. The article reports the development process of this robot named Mon-chan (a monster-looking doll bringing harmonious communications among users), and presents results of an evaluation study conducted in the nursing home, exploring future research agendas.

## Research Settings, Robot Design and Development Process

The development process started from users’ perspectives (older persons and care professionals), extracting the core issues identified with the previous setup and using various assistive technologies (a monitoring camera and communication robots, in particular). The gemba principle drove this process with frontline care professionals representing the voice of care recipients, particularly those who are not physically mobile enough to reach and press the nurse call button when they need assistance.

### Settings and Participants

In Japan, there are different types of care facilities. The facility selected for this study is a special nursing home for older people. There are 30 residents (25 female, 5 male; $86.8 \, \pm 6.8$ years old). From the group of care professionals, 4 senior staff members in the nursing home participated in the design process of the robot and the data collection. The years of experience vary from 7 to 20 (11 on average) years. In order to ensure objectivity, the analysis was conducted by three researchers who were not involved in the collection of data. Ethics approval was granted by the Social Welfare Corporation Tokyo Sacramental Ethics Committee (TS 2019- 004). Consent was sought from each participant and their family. The research was conducted between October 2019 and March 2020.

### User-Centered Design and Development in a Nursing Home

Most conventional communication robots have a hard shell which gives a cold impression for both residents and staff. As suggested by previous research, the sense of touch is a critical element for older adults’ engagement with social robots [5, 6, 16, 24–26]. The hard shell also meant that physical deployment is highly restricted in the context of nursing homes, as it requires a solid surface, such as a bedside table. As the physical distance between SARs and care recipients was not adjustable, residents had difficulty hearing and understanding what SARs were saying. From the staff’s point of view, there has always been an issue of having to go to the room to see what has happened to a resident each time a nurse call button was pressed, particularly during nighttime. It was hoped that a work system, aided by ATs, could send the warning signs or visual information wherever care professionals are, so that they can arrive in time before problems such as falls occur. In the past, there were some weaknesses in the ICT environment issues, such as time lag via the Cloud and malfunctions.

**Fig. 1:**
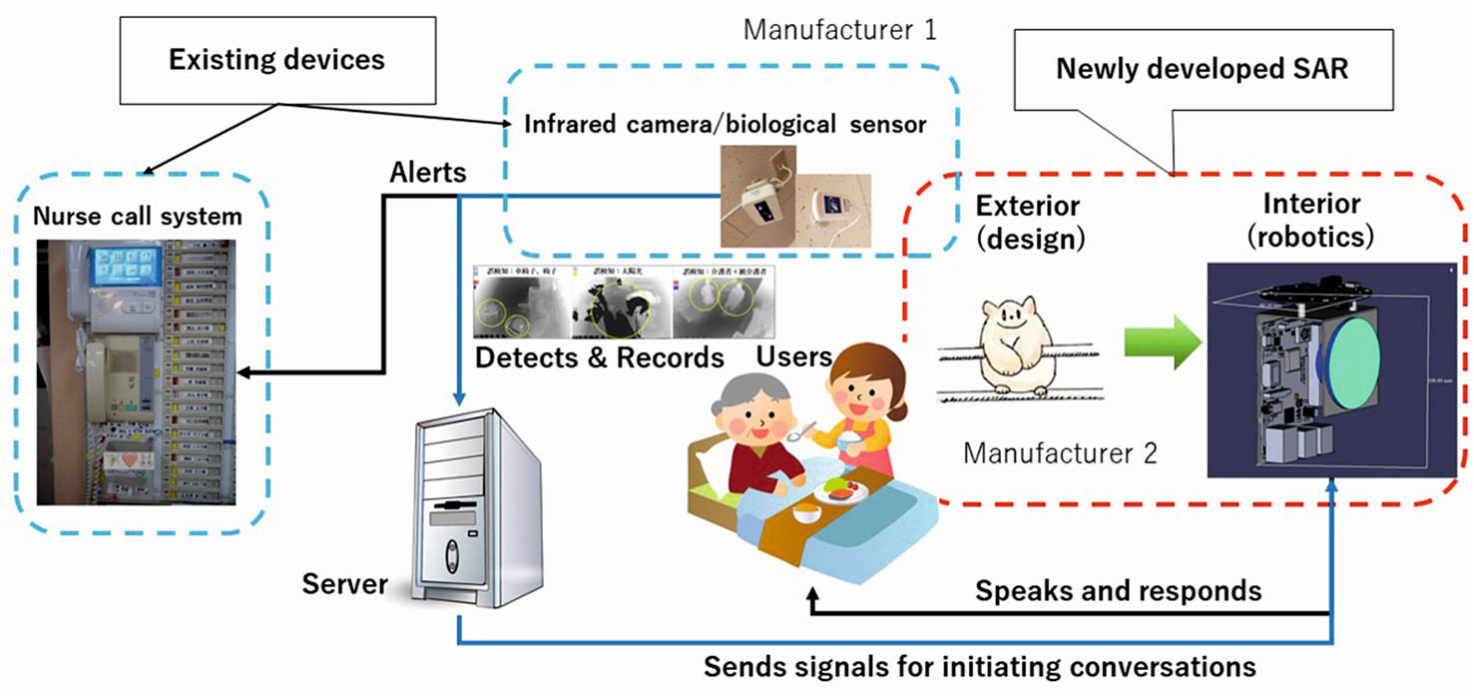
Overall concept and SAR development

User-centered design dictates that priority should be placed on development of a communication robot that meets their needs. The overall concept therefore was to create a soft shell SAR with an input/output device which is connected to the nurse call button and the monitoring camera. Figure 1 describes the overall concept and the development process.

The system requirements include the capability of (i) remotely monitoring and sending signals to care staff when help is needed; (ii) watching over older people around the bed; and (iii) providing older people with a voice-controlled alternative method to a nurse call; and (iv) small, compact and audible robot with soft and warm appearance, which can be securely hung over the bed-rail without hindering its vertical movement.

The first two requirements ((i) and (ii)) were fulfilled by the existing devices, which monitor the person with a bed-side infrared camera. In case of emergencies such as falls, alerts were sent to the central nursing station as well as care professionals on duty. For the third requirement (iii), a voice-controlled alternative method was considered at the beginning. However, the idea of developing and installing a two-way communication system into the robot had to be rejected for reasons of cost and time. Instead, the team decided to rely on the existing nurse call system, and this was activated when the alert was received by care staff, so that the care recipient in need of help could communicate verbally. The main focus of this project was narrowed down to fulfil (iv).

**Fig. 2:**
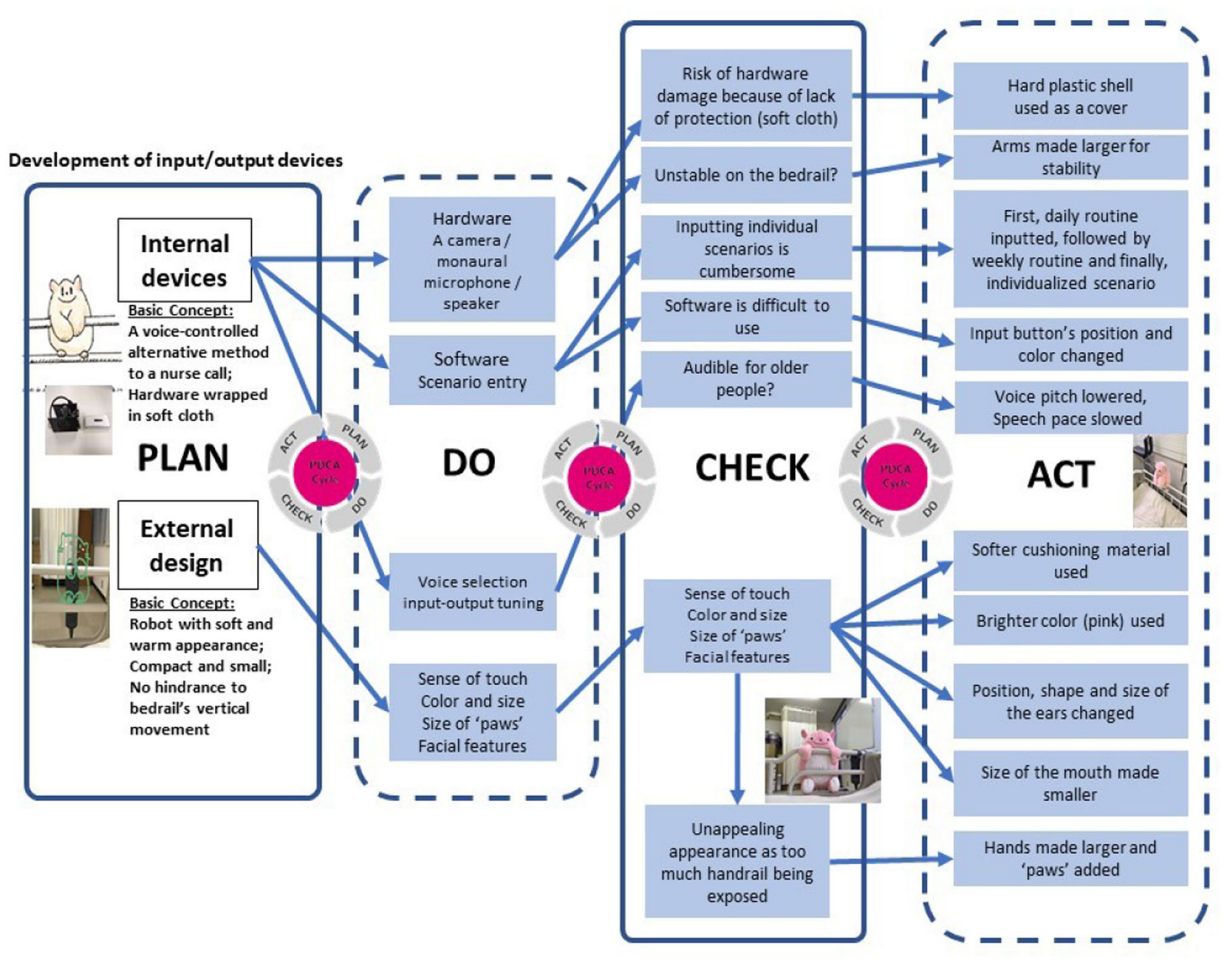
Two-stage process of developing SAR “Mon-chan” using the PDCA method

From basic design to development of the interface design, the iterative, PDCA (plan, do, check, do, act) cycle method was applied (Fig. 2). The processes for the robot (exterior) design and internal functional design were separated, and the latter proceeded with the development of verbal communication functions (e.g. system design and scenarios for conversations) and non-verbal aspects.

Much attention was paid to the human–robot interface design, with consideration of its appearance and voice functionality. At each stage, discussions were held between manufacturers and frontline care professionals. As a result, softer plush fabrics were chosen, and the SAR made more compact, yet more stable on the bedrail using larger paws, though it was not feasible to add a variety of voice types. Table 1 shows the changes made to the design and functions of the SAR after the iterative process of consultations.

**Table 1:** Intended design and function of SAR “Mon-chan” and end product

	Intended design and function	End product
Design	Soft and warm; appealing appearance; compact; stable on bedrail	Plush fabrics used; smaller mouth/ ears; larger paws for ease of hanging
Function	Audible sound (tone and pace); flexibility in changing scenario type of voice; device protected from vibrations (e.g. substrate)	Lower pitched voice; slower pace of speech; new colored record button; individualized scenarios; substrate covered with plastic

**Table 2:** Three types of communication scenarios

Scenario common to all individuals	Scenario unique to	Conversations activated by monitoring camera
e.g. “Soon breakfast	e.g. “Today you’ll	e.g. “What’s happened?
will be served.”	have a bath” “Would	Would you like to go to
(6:30 am), ‘Soon it’ll	you like to join	the toilet?”, “Staff
be time for physical	today’s calligraphy	will be here in a minute,
exercise” (11 am). “Did	class, starting soon?”,	so please wait a
you have a good day?	“Today you have an	moment.”, “How are you
Please have some good	appointment with your	today?”
rest” (8 pm)	doctor.”	

### Development of Conversation Scenarios

One of the key aspects of the SAR design was the set of conversation scenarios, which were developed based on daily care routines (see Table 2). Messages about meals, wake-up, and bedtime (the timing of wake-up and bedtime messages were set individually) was input for all residents. A conversation scenario about recreation and toileting was tailored for each person, reflecting their lifestyles and daily patterns. In addition, the team enabled the scenario to be adjusted for each person, based on previous recordings captured by the monitoring camera. This was particularly important for conversations taking place during the night, when residents wake up. When the movement of a resident in her room is detected, the signal sent from the monitoring sensor (installed in each room) instructs the SAR’s server to speak, and based on the created scenario, recorded voice and sound synthesis are transferred via the SIP server, and the SAR speaks to the resident. For example, the Mon-chan robot asks “What’s happened?” when the resident tries to get up. In response to her saying “I want to go to the toilet”, Mon-chan says “Staff will be here in a minute, so please wait a moment.”

**Fig. 3:**
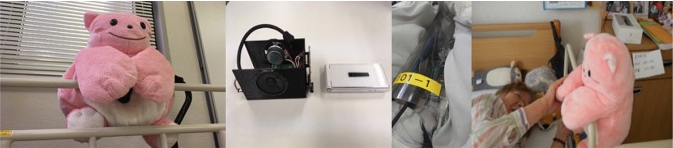
SAR “Mon-chan” and equipment inside the robot

Mon-chan was produced this way, and this communication robot (10 [cm] $\times$ 7 [cm] $\times$ 5 [cm]), which looks like a stuffed animal, contains an I/O device (Table 3, Fig. 3).

**Table 3:** Features of SAR Mon-chan

Input/output device	A camera/monaural microphone/speaker/power over Ethernet (POE) available
Interactions	Typed sentences converted into voices. Voices pre-programmed and personalized according to the needs of each participant
Features	Provides safety monitoring via infrared sensor and alert functions, connected to nursing station. Allows users to speak with a care professional through the device

## Evaluation of Verbal and Non-verbal Mon-chan

As previously mentioned, Mon-chan is a communication robot that has soft appearance and pleasant touch, resembling a non-verbal pet-type robot. When it is used by the bedside, it is also linked with an infrared monitoring camera and a biological sensor.

### Evaluation Methods and Procedures

For this evaluation, we employed two methods (the interRAI and Video Coding Protocol-Incorporating Observed Emotion (VC-IOE)). Verbal Mon-chan was used at all times in each resident’s room for all participants, while Mon-chan, in both verbal and non-verbal mode, was tested when the latter method was used to observe each resident’s personal interactions with Mon-chan. The evaluation took place for 4 weeks during the months of February and March 2020. The video-recording and observation sessions were held in the fourth week. The interRAI is one of the standardized assessment instruments for those who receive care. These scales measure physical abilities (activities of daily living (ADLs)), cognitive impairment and quality of life. The assessment items are organized into sections dealing with issues such as cognitive patterns, communication and hearing patterns, and physical functionality [27–29]. The self-rated or assessor-rated interRAI scores can be used as proxy for older people’s Quality of Life (QoL). Prior to the introduction of Mon-chan, we collated residents’ scores for two categories (E: Mood and Behavior; F: Psychosocial well-being) [30], and compared them with those collected 4 weeks after the introduction of Mon-chan (Table 4). The higher the scores are, the larger the burden is on mood, behavior and psychosocial well-being. The results are presented below (Sect. 3.2).

**Table 4:** Excerpt of interRAI (E: Mood and Behavior, F: Psychosocial well-being) [30],?

	SECTION E. MOOD AND BEHAVIOR
E1.	INDICATORS OF POSSIBLE DEPRESSED, ANXIOUS, OR SAD MOOD
	Code for indicators observed in last 3 days, irrespective of
	the assumed cause [Note: Whenever possible, ask person]
	0. Not present; 1. Present but not exhibited in last 3 days;
	2. Exhibited on 1–2 of last 3 days; 3. Exhibited daily in
	last 3 days
E1a.	Made negative statements
E1b.	Persistent anger with self or others
E1c.	Expressions, including non-verbal, of what appear to be unrealistic fears
E1d.	Repetitive health complaints
E1e.	Repetitive anxious complaints/concerns (non-health-related)
E1f.	Sad, pained, or worried facial expressions
E1g.	Crying, tearfulness
E1h.	Recurrent statements that something terrible is about to happen
E1i.	Withdrawal from activities of interest
E1j.	Reduced social interactions
E1k.	Expressions, including nonverbal, of a lack of pleasure in life
E3.	BEHAVIOR SYMPTOMS
	0. Not in last 3 days; 1. Not in last 3 days, but often feels that way;
	2. In 1–2 of last 3 days; 3. Daily in last 3 days
E3a.	Wandering
E3b.	Verbal abuse
E3c.	Physical abuse
E3d.	Socially inappropriate/disruptive behavior
E3e.	Inappropriate public sexual behavior or public disrobing
E3f.	Resists care
E3g.	Absconding or at risk of absconding
	**SECTION F. PSYCHOSOCIAL WELL-BEING**
F3.	CHANGE IN SOCIAL ACTIVITIES IN LAST 90 DAYS
	0. No decline; 1. Decline, not distressed; 2. Decline, distressed
F4.	LENGTH OF TIME ALONE DURING THE DAY (MORNING
	AND AFTERNOON)
	0. Less than 1 h; 1. 1–2 h; 2. More than 2 h but less than 8 h;
	3. 8 h or more
F5.	WILLINGNESS TO INITIATE OR PARTICIPATE
	0. Not in last 3 days; 1. Not in last 3 days, but often feels that way;
	2. In 1–2 of last 3 days; 3. Daily in last 3 days
F5a.	At ease interacting with others
F5b.	At ease doing planned or structured activities
F5c.	Accepts invitations to most group activities
F5e.	Initiates interaction(s) with others
F5f.	Reacts positively to interactions initiated by others
F5g.	Adjusts easily to change in routine
F6.	INSTABILITY OF INTERPERSONAL RELATIONSHIP
	0. No; 1. Yes
F6a.	Conflict with or repeated criticism of other care recipients
F6b.	Conflict with or repeated criticism of staff
F6c.	Staff report persistent frustration in dealing with person
F6d.	Family or close friends report feeling overwhelmed by person’s illness

**Table 5:** VC-IOE items adapted for Mon-chan [31]

Emotion	Positive	Smiling, laughing, singing, responding to Mon-chan
(Facial emotional response)	Negative	Physical aggression, yelling, cursing, drawing eyebrows together. Clenching teeth, pursing lips, narrowing eyes. Voice shaking, shrieking, repetitive calling out, line between eyebrows. Lines across forehead, tight facial muscles. Crying, frowning, eyes drooped, moaning, sighing, eyes/head turned down
	Neutral/missing	Relaxed or no sign of discrete facial expression
Verbal engagement	Positive	General talking. Participating and maintaining conversation, verbally responding to statements/questions. Expressing positive feelings towards Mon-chan
	Negative	Verbalizes the desire to leave. Refuses to participate in the activity by verbalizing “no”, “stop”, etc. Makes repetitive generalized somatic complaints. Cursing and swearing
	Neutral/missing	Not participating in or maintaining conversation. Not responding or talking to the facilitator when prompted
Auditory engagement	Positive	Participating and maintaining conversation
	Negative	Hard of hearing, no response
Visual engagement	Positive	Appears alert, and maintaining eye contact with facilitator or others. Eyes following facilitator or others
	Negative	Appears inattentive, blank stares into space, no eye contact.
Behavioral engagement	Positive	Touching or attempting to touch Mon-chan. Stroking, petting, nuzzling
	Negative	Hitting, shaking and handling Mon-chan inappropriately. Pushing Mon-chan away
	Missing	No touching; no physical contact with Mon-chan or not handling Mon-chan
Collective engagement	Yes	Encouraging others to interact with Mon-chan. Introducing Mon-chan to the facilitator. Using Mon-chan as a communication channel to interact and talk with others
	No	No sign of collective engagement
Evidence of agitation	Yes (verbal, vocal, motor activity)	Restlessness, repeated/agitated movement (frequent non-purposeful movement), moving in chair, picking at and fiddling with clothes; repetitive rubbing of own limbs or torso; appears anxious, abusive or aggressive toward self or others
	No	No sign of agitation as described above
Need of facilitator’s involvement	No	Only at the beginning
	Yes	Prompting 2–3 times
		Prompting several times

**Fig. 4:**
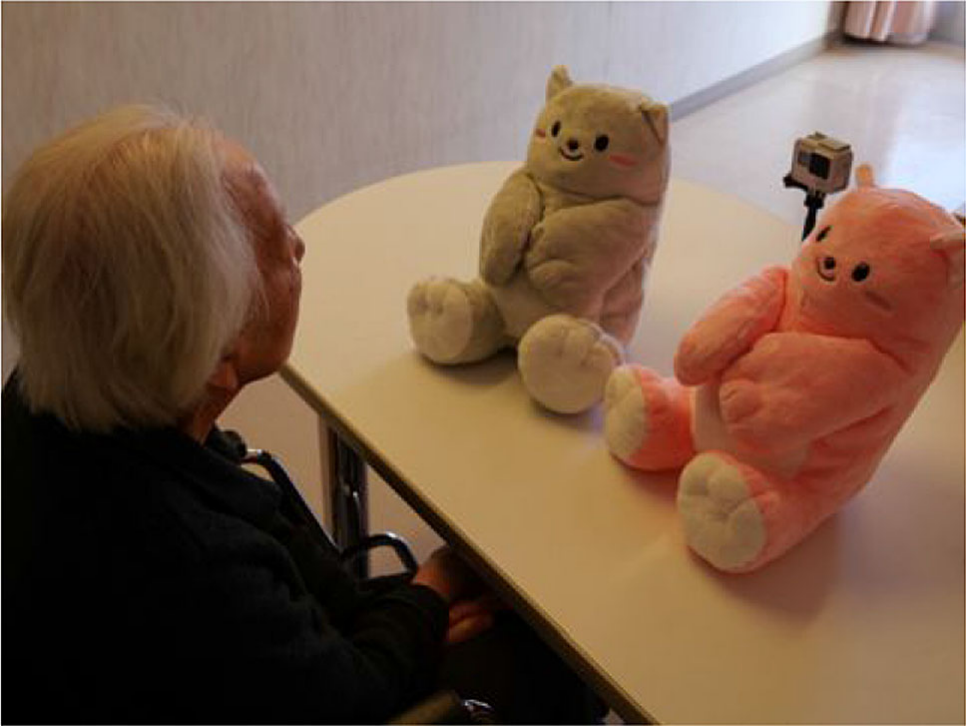
Verbal exchanges with Mon-chan during recreation

The other method, VC-IOE was devised by Jones et al. [32] for observing short-term emotional changes when older persons use the pet-type robot PARO. The list of observation items include six categories “Emotional engagement”, “Verbal engagement”, “Visual engagement”, “Behavioral engagement”, “Collective engagement” and “Agitation”). This coding framework was adapted and translated into Japanese [ [32], p.380], and used for the current study (Table 5). The participants were asked to spend time with Mon-chan individually during their recreation, and their 3-min interactions were video-taped with their permission (Fig. 4). A pair of care professionals observed the interactions and took notes. This was carried out partly to test individual responses to Mon-chan, which is designed to be both a non-verbal (cuddly toy) and a verbal robot. Mon-chan in both modes was examined. Three researchers (two care professionals and one external researcher) subsequently analyzed the recorded videos, using the VC-IOE method (Japanese version). The disagreements between coders were adjusted after a few pilot recordings and discussions. The results are shown in Sect. 3.3.

### Changes of QoL Assessed by interRAI

The overall interRAI scores for the two categories were $11.9 \pm 9.7$ before the introduction of Mon-chan. Four weeks later, the scores changed to $10.2 \pm 7.9$, which was statistically significant ($p <0.01$). At the individual level, 57 percent of participants’ scores improved while 20 percent remained the same and 23 percent worsened. In particular, E1 “Indicators of possible depressed, anxious or sad mood” showed a marked improvement ($p <0.005$), while E3 “behavior and mood” showed some improvements ($p=0.08$). Looking at each item, E1b (persistent anger with self or others, $p = 0.03$), E1d (repetitive health complaints, $p = 0.02$), E1e (repetitive anxious complaints/concerns (non-health related), $p = 0.02$), E1h (recurrent statements that something terrible is about to happen, $p = 0.03$), these individual items for category E also indicated statistically significant improvement ($p <0.05$). Furthermore, positive improvements were also found in E3b (Verbal abuse, $p = 0.08$), E3e (Inappropriate public sexual behavior or public disrobing, $p = 0.08$) and E3g (Absconding or at risk of absconding, $p = 0.08$). On the contrary, F4 (Length of time alone during the day, $p = 0.39$), F5 (Willingness to initiate or participate, $p = 0.4$) and F6 (Instability of interpersonal relationship was statistically significant, $p = 0.33$) did not show any change. Overall the psychosocial burden was reduced, and it can be argued that Mon-chan had a positive effect on the quality of life of the participants.

### Short-Term Emotional Changes Captured by VC-IOE

**Fig. 5:**
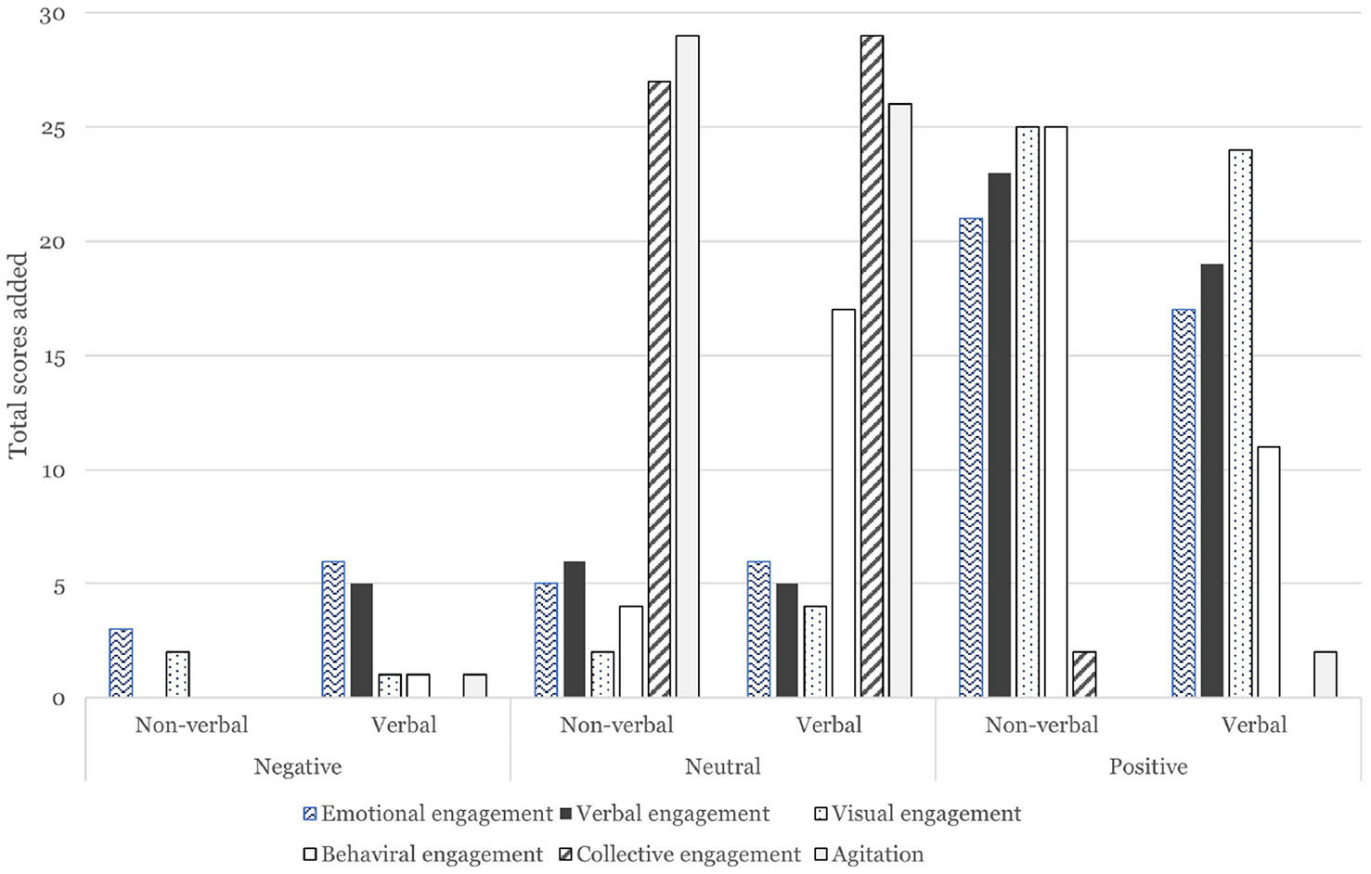
Verbal and non-verbal Mon-chan compared

For this study, each item was given a score of − 2 (worse), − 1 (bad), 0 (no change), + 1 (good), + 2 (improvement) by the coder, observing the three-minute clip. Although it was a short-term change, a positive stimulating effect was observed for the categories (“Emotional engagement”, “Verbal engagement”, “Visual engagement”, “Behavioral engagement”). Figure 5 shows the comparison of total scores in each category between verbal and non-verbal Mon-chan. The results demonstrate particularly positive engagement with the (non-verbal) Mon-chan doll compared to that with the verbal Mon-chan robot. However there was no statistically significant difference. The combined counts of positive scores (“good” and “improvement”) accounted for more than half (55.2%) for non-verbal Mon-chan, and 42.0% for verbal Mon-chan (Fig. 5). We further investigated the data for non-verbal Mon-chan’s effect on the participants with dementia.

**Fig. 6:**
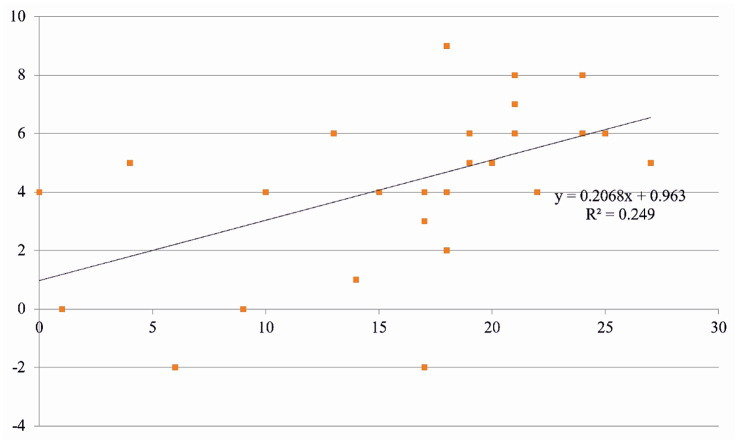
Relationship between non-verbal Mon-chan’s effect and the level of dementia (X axis: MMSE scores; Y axis: individual scores using the VC-IOE method)

Focusing on non-verbal responses, we used the Mini-Mental State Exam (MMSE) scores to measure the level of dementia, as it was deemed pertinent to examine whether any patterns were detected between those scores and the results from the VC-IOE method. The MMSE is a 30-point test used to measure the degree of cognitive ability. The test includes orientation to time and place, short-term memory, attention and ability to solve problems, language and comprehension and motor skills, and the greater the scores are, the greater the ability is [31]. A mildly positive linear regression was observed (Fig. 6), suggesting that there is a possible positive pattern depending on the level of dementia. People with dementia can potentially make positive use of the Mon-chan doll. As previous studies show, even soft plush toys without communicative functions can have therapeutic impact on older people with impaired cognitive ability, with the assistance of carers.

## Discussion

The research team aimed to develop an SAR which is user-friendly, is appealing, and contributes to better and safer care delivery. The aim was to overcome formerly observed weaknesses of SARs and draw on the views of users (residents and care professionals working on site) in the nursing home. The result was the production of Mon-chan, which was designed, developed, introduced and evaluated on site. User-centered design still remains relatively uncommon in the domain of robotics design and development for care of older people, particularly for SARs in Japan [6–8]. This can be attributed to several factors, including the difficulty of having a regular dialogue or forum where developers, care recipients and care professionals can meet and discuss their needs and preferences. Moreover, there has been a scarcity of research evidence to support the arguments that these SARs can improve quality of life for older adults. Because of this, stakeholders and users can be skeptical and take a cautious approach [33]. In the last few years, more research results are being published [4, 17–20], and this situation is likely to improve. There are also some studies highlighting (potential) users’ perceptions and attitudes towards care robots [34, 35]. Ethical issues have also been examined and considered by researchers and bodies such as the IEEE [36–38]. Furthermore, SARs can only be utilized fully when care recipients and care professionals find them both user-friendly and useful. The application of ATs in this area needs careful consideration of users’ needs and workforce development implications. Successful implementation requires multi-level thinking (from user experience and organizational processes to policy and industry context) and the understanding that technology-supported work is cooperative and embedded in organizational routines [39].

For this study, we also assessed the changes in interRAI scores over the 4-week period when Mon-chan was used. The results indicated that mood and behavior of the participants improved significantly, while psychosocial aspects remained stable. Simultaneously, using the VC-IOE observation framework, the findings showed positive engagement of the participants, irrespective of the difference in type (verbal vs. non-verbal). Non-verbal use of Mon-chan exceeded the expectations of this study, generating very positive engagement in emotional, verbal, visual and behavioral aspects (Fig. 5). While the mechanism through which the technology-driven system improved residents’ quality of life requires future study, it could be argued that the reflection of their voices in the design process of Mon-chan contributed to their engagement. On the other hand, the positive impact of Mon-chan with communicative functions was not clear, considering the relatively lower positive scores than those for non-verbal Mon-chan (Fig. 4). Although the improved sound quality of Mon-chan’s verbal function was noted, the interactions between care recipients and verbal Mon-chan did not yield positive behavioral or collective engagement. Therefore, as indicated by the previous study [40], the impact of verbal SARs and non-verbal plush toys on older adults with dementia is inconclusive. Further research is needed to examine the relationship and improve the communication functionalities. It is also worth investigating how we can accurately measure the impact of these SARs and ATs on the quality of life of older people. There are a variety of assessment tools such as the WHO’s International Classification of Functioning, Disability and Health (ICF), Dementia Behavior Disturbance Scale-13 (DBD-13), Cohen-Manfield Agitation Inventory (CMAI), Neuropsychiatric Inventory with Caregiver Distress Scale (NPI-D), with the interRAI being merely one of them. For our study, interRAI was deemed highly effective and accurate in capturing the changes in residents’ mood and behavior, though this merits further examination.

The limitations of this study include the small sample size and the absence of a longitudinal study design to examine the impact of a longer period of SAR use on care recipients, care professionals and the nursing home. The analysis of contributing factors such as organizational context, physical environment, and the impact of unanticipated disruptions to daily care routines (e.g. Covid19 pandemic) is also worth considering, although it is beyond the scope of this paper. Nonetheless, the study demonstrated the significance of care professionals’ involvement in the design and development of an original soft social robot as a way of translating the voice of older adults with impaired cognitive and sensory ability. By assessing the impact, and despite some inconclusive results, the production loop of SARs was closed, which fulfilled the principle of gemba. Whether care professionals’ involvement in SARs design, production and evaluation can lead to greater job satisfaction and a better work environment also needs further research. However, this study is well-aligned with the progress towards the RRI framework [9], with greater public and patient involvement. Future research into and development of care robots need to examine the connection between process and outcomes.

## Conclusions

This study outlined the user-centered design and evaluation of a socially assistive robot within the context of a nursing home in Japan. The collaborative developmental process among different stakeholders-manufacturers, care professionals, care recipients and researchers, improved quality of life among residents. The original SAR “Mon-chan” was connected to existing ATs (monitoring camera, nurse call, and biomedical sensor), which contributed to safety and enhanced quality of care. This safety function, in addition to the socially assistive one, provides another layer of security for care recipients and care professionals. As the new coronavirus infectious disease has highlighted, there is now a new focus on the introduction of telemedicine, and applications of remote monitoring and care delivery models as countermeasures for the spread of the virus and the protection of older people in nursing care facilities. The importance of user-oriented design and development of robotics-aided care for the improvement of a care system will continue to be highlighted in the future.
